# P-1096. Activity of Ibrexafungerp and Comparator Antifungals Tested against *Candida* and *Aspergillus* Isolates Collected from Invasive Infections in a Global Surveillance Program in 2023

**DOI:** 10.1093/ofid/ofae631.1284

**Published:** 2025-01-29

**Authors:** Marisa Winkler, Paul Rhomberg, Abigail Klauer, Samuel Edeker, Sharon Min, Mariana Castanheira

**Affiliations:** Element Materials Technology/Jones Microbiology Institute, North Liberty, Iowa; Element Materials Technology/Jones Microbiology Institute, North Liberty, Iowa; Element Materials Technology/Jones Microbiology Institute, North Liberty, Iowa; Element Materials Technology/Jones Microbiology Institute, North Liberty, Iowa; GlaxoSmithKline, Collegeville, Pennsylvania; JMI Laboratories, North Liberty, Iowa

## Abstract

**Background:**

Ibrexafungerp (IBX) is a novel triterpenoid antifungal agent that was approved by the U.S. FDA for the treatment of vulvovaginal candidiasis (VVC) in 2021. Further studies are ongoing to assess the efficacy of IBX for the treatment of invasive candidiasis (IC) and other refractory fungal infections, including aspergillosis. CLSI, EUCAST, or FDA clinical breakpoints or epidemiological cutoff values are not yet defined for IBX against fungi. We evaluated the activity of IBX and comparator agents against clinical isolates from IC and invasive aspergillosis infections collected from a global surveillance program in 2023.

MIC50/90 (mg/L) for ibrexafungerp (IBX) and comparator agents against 5 most common Candida spp., C. auris, and 3 most common Aspergillus spp. in 2023 SENTRY Antifungal Surveillance Program

**Figure:**
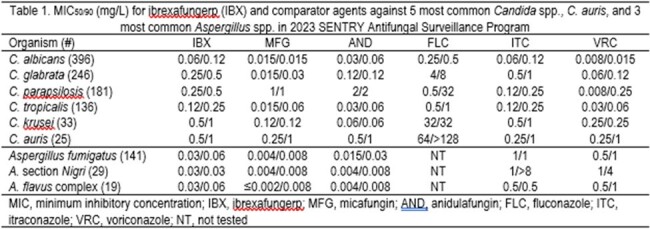

**Methods:**

A total of 1120 *Candida* spp. and 204 *Aspergillus* spp. isolates consecutively collected as part of the SENTRY Program were evaluated. Isolates were recovered from 11 medical centers in North America, 18 in Europe, 7 in Asia-Pacific, and 5 in Latin America. Identification was performed by MALDI-TOF MS and/or sequencing and susceptibility testing was performed using the CLSI broth microdilution method for IBX, anidulafungin (AND), micafungin (MFG), fluconazole (FLC), itraconazole (ITC), and voriconazole (VRC).

**Results:**

Table 1 displays MIC_50/90_ results for the 5 most common *Candida* spp., *C. auris*, and the 3 most common *Aspergillus* spp. The IBX MIC_50/90_ results were low against all organisms including organisms of critical concern like *C. auris*. Among *Candida* species, IBX had similar or higher MIC_50/90_ to AND except for *C. parapsilosis* where IBX MIC_50/90_ values were 4- to 8-fold lower. MFG was more potent (4- to 16-fold) against most *Candida* spp. except for *C. parapsilosis* where IBX had 2-to 4- fold lower MIC_50/90_ and *C. auris* where IBX was similar to MFG. Notably, the highest MIC result observed for IBX against *C. auris* was 1 mg/L. MIC_50/90_ values for IBX were also 8- to > 256-fold lower than azoles against *Aspergillus* spp. but higher (2- to ≥ 16-fold) than MFG or AND.

**Conclusion:**

IBX has good activity against the predominant *Candida* and *Aspergillus* species from a worldwide contemporary collection. This agent may represent an oral option for the management of difficult-to-treat invasive fungal pathogens such as *C. glabrata*, *C. auris*, and *Aspergillus* spp. from diverse infection sources in addition to its current indication for VVC.

**Disclosures:**

**Marisa Winkler, MD, PhD**, Element Iowa City (JMI Laboratories) was contracted to perform services in 2023 for > 30 biotech and pharmaceutical companies: Grant/Research Support **Sharon Min, MS**, GSK: Employee

